# A case of malignant lymphoma of the extrahepatic bile duct diagnosed by detailed imaging examination and endoscopic ultrasound-guided fine needle aspiration

**DOI:** 10.1007/s12328-024-02075-x

**Published:** 2024-11-30

**Authors:** Noriaki Iijima, Shinya Nakamura, Yasutaka Ishii, Yumiko Tatsukawa, Juri Ikemoto, Sayaka Miyamoto, Kazuki Nakamura, Masaru Furukawa, Koji Arihiro, Shiro Oka

**Affiliations:** 1 Department of Gastroenterology, Graduate School of Biomedical and Health Sciences, Hiroshima University, 1-2-3 Kasumi, Minami-Ku, Hiroshima, 734-8551 Japan; 2 Department of Pathology, Hiroshima University Hospital, Hiroshima, Japan

**Keywords:** Malignant lymphoma, Diffuse large B-cell lymphoma, Bile duct, Endoscopic retrograde cholangiopancreatography, Endoscopic ultrasound-guided fine-needle aspiration

## Abstract

A 70-year-old woman presented to our hospital with abdominal pain. Imaging examinations showed diffuse and extensive wall thickening at the perihilar bile duct; however, the degree of stricture was mild, and the mucosal epithelium was smooth. A transpapillary biopsy was performed considering cholangiocarcinoma and IgG4 sclerosing cholangitis as differential diagnoses; however, no pathologic diagnosis was obtained. Peroral cholangioscopy revealed a regular epithelium at the stricture, and endoscopic ultrasound-guided fine-needle aspiration (EUS-FNA) of the enlarged lymph node confirmed the diagnosis of diffuse large B-cell lymphoma. Multiagent chemotherapy was administered, which led to complete remission. Because primary bile duct malignant lymphomas are rare and specific, clinical, and imaging findings are lacking, and many of those reported so far have been diagnosed by postoperative pathology. As chemotherapy is the first-line treatment for malignant lymphoma, obtaining an accurate diagnosis is crucial. Our findings support that smooth and mild biliary strictures with mainly submucosal wall thickening may be characteristic imaging findings of primary bile duct malignant lymphoma, and that peroral cholangioscopy and EUS-FNA may be helpful for an accurate diagnosis.

**Supplementary Information:**

The online version contains supplementary material available at 10.1007/s12328-024-02075-x.

## Introduction

Malignant lymphoma (ML) is known to occur not only in the lymph nodes but also in various other organs, the most common extranodal organs of which are the gastrointestinal tract lesions [1]. ML causes bile duct stricture in 1–2% of all cases [2]; however, the main cause of stricture is extrinsic invasion of adjacent organs or compression by peribiliary lymph nodes, and primary bile duct ML is rare. Owing to its rarity, the imaging findings of bile duct ML are not readily available. Moreover, obtaining a sufficient amount of tissue via biopsy is a challenge [3, 4]. Therefore, the accurate diagnosis of bile duct ML is challenging, and many of the reported cases were resected with a diagnosis of cholangiocarcinoma and diagnosed as ML postoperatively [4–7]. Herein, we report a case of ML of the extrahepatic bile duct diagnosed using detailed imaging examination and endoscopic ultrasound-guided fine needle aspiration (EUS-FNA).

## Case report

**Fig. 1:**
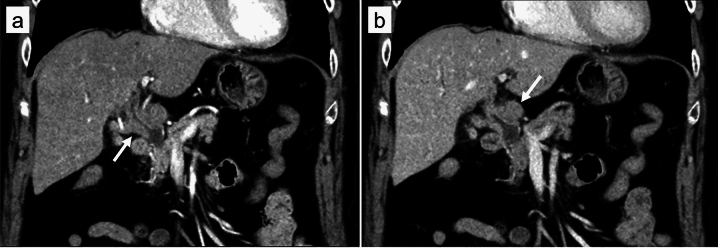
Contrast-enhanced computed tomography. **a** Image depicting thickening of the perihilar bile duct wall with contrast enhancement (arrow). **b** Image of an enlarged lymph node next to the perihilar bile duct (arrow)

**Fig. 2:**
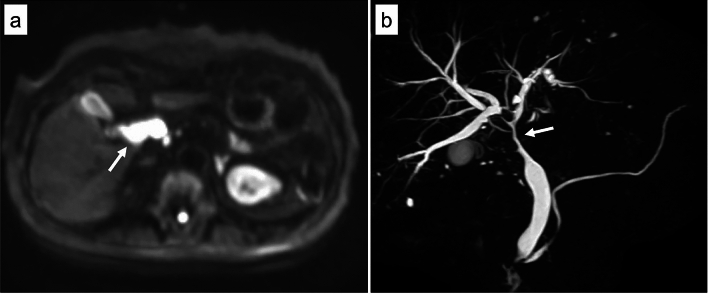
Magnetic resonance imaging. **a** The thickened wall of the perihilar bile duct and adjacent mass shows a hyperintense signal on diffusion-weighted imaging (arrow). **b** Magnetic resonance cholangiopancreatography reveals stricture of the bile duct from the hepatic hilum to the vicinity of the confluence of the cystic duct (arrow), and the upstream bile duct is slightly dilated

**Fig. 3:**
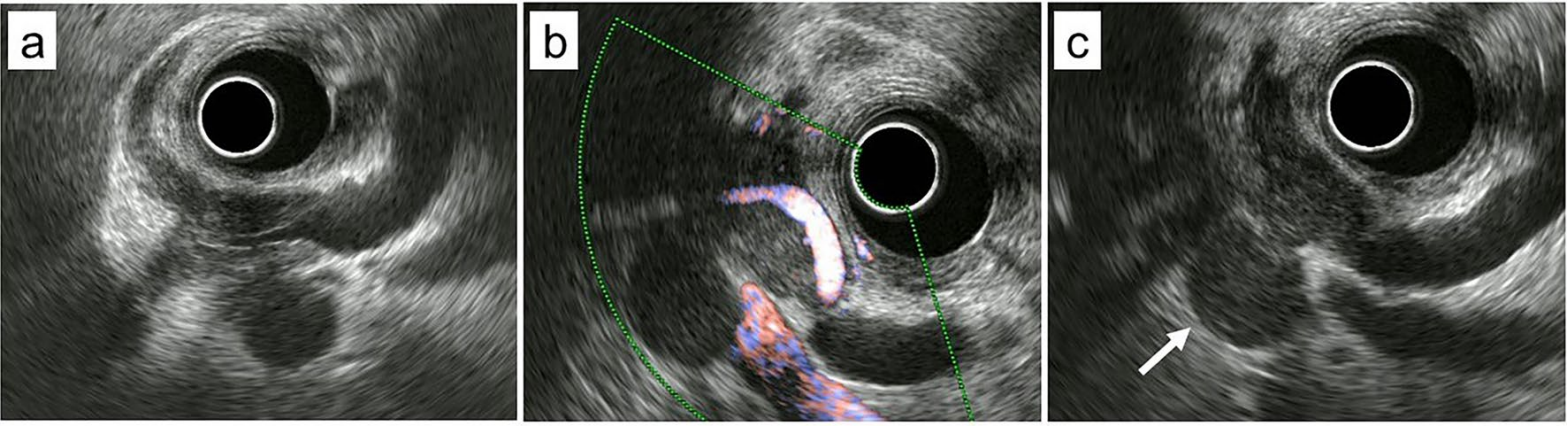
Endoscopic ultrasonography. **a** The thickened bile duct shows a smooth inner border echo. **b** The lumen of proper hepatic artery penetrating the thickened wall is preserved. **c** A 2-cm mass (arrow) is found close to the lesion, which is considered an enlarged lymph node. The boundary between the bile duct and the mass is clear and no continuity is observed

**Fig. 4:**
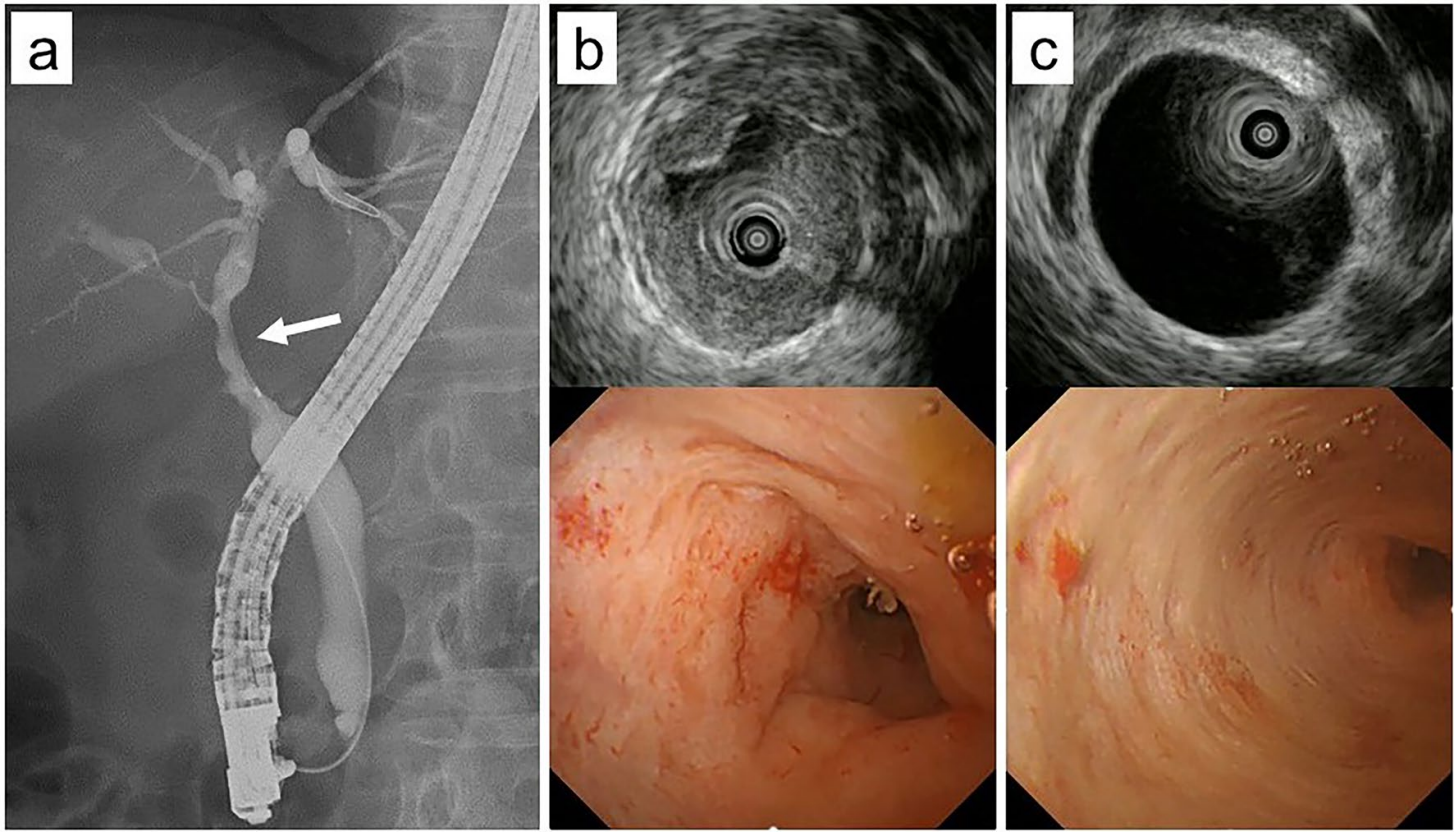
Endoscopic retrograde cholangiopancreatography. **a** A relatively extensive, gently rising stricture is observed in the perihilar bile duct (arrow). **b** In the stenotic area, intraductal ultrasonography reveals a homogeneous wall thickening of 3.5 mm and peroral cholangioscopy shows submucosal tumor-like-circumferential narrowing with mildly irregular and uneven but smooth mucosa, and no severe mucosal irregularities or unevenly dilated blood vessels suggestive of cholangiocarcinoma were observed. **c** In the non-stenotic area, intraductal ultrasonography reveals no wall thickening and peroral cholangioscopy shows no mucosal irregularity

**Fig. 5:**
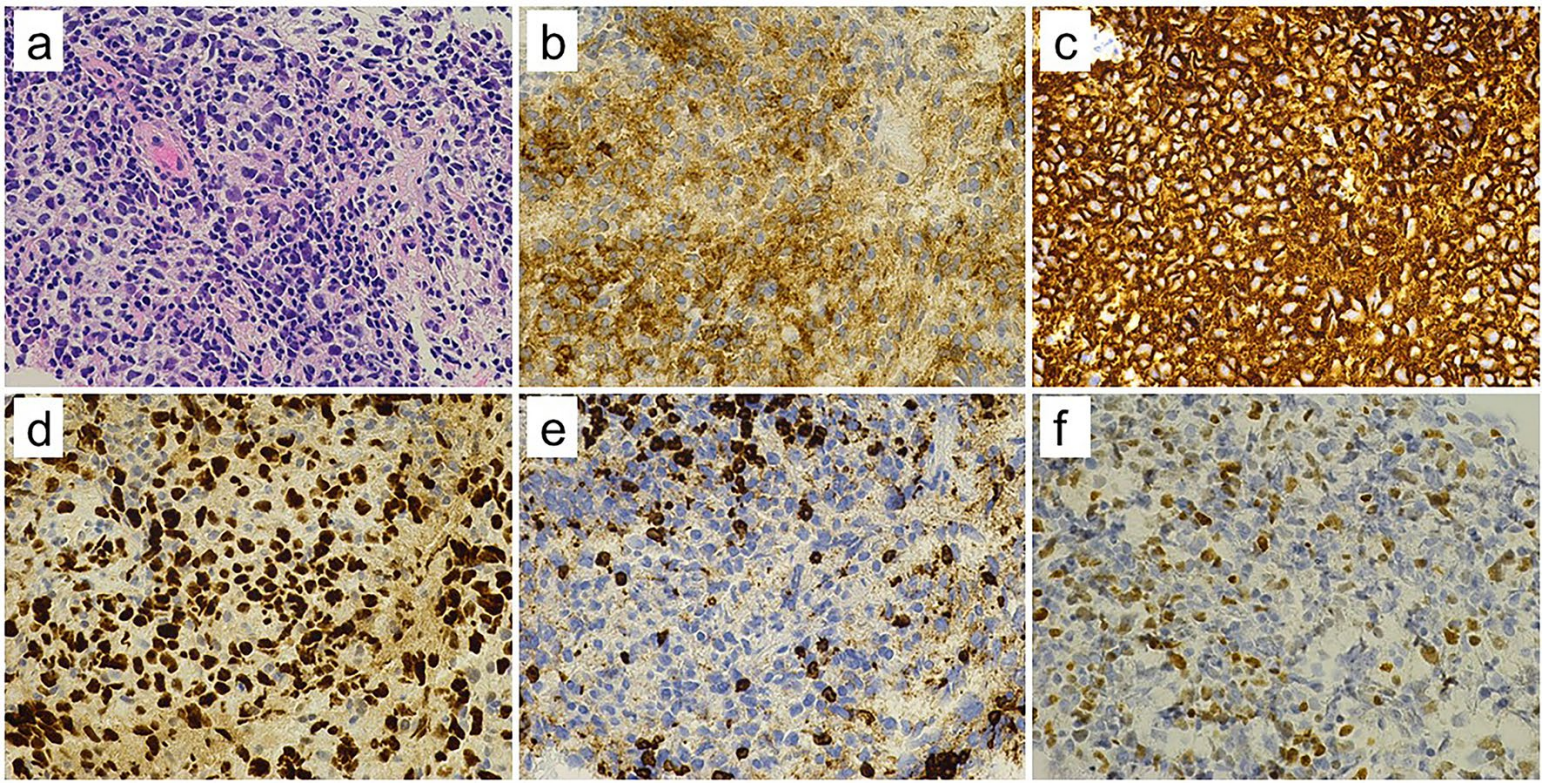
Pathologic findings of endoscopic ultrasound-guided fine needle aspiration specimens. A diffuse proliferation of large, atypical cells with anisotropic, irregular nuclei is observed by hematoxylin and eosin staining (**a**). Immunohistochemical staining is positive for CD10 (**b**), CD20 (**c**), and BCL-6 (**d**) and negative for CD3 (**e**) and MUM-1 (**f**), consistent with diffuse large B-cell lymphoma, germinal center B-cell–like subtype

A 70-year-old woman presented to our hospital complaining of epigastric pain for one month. She had a medical history of hypertension, arterial fibrillation, bronchial asthma, and chronic renal failure. She had no specific family history or history of alcohol consumption or smoking. Physical examination revealed no jaundice, and no superficial lymph nodes were palpable. Laboratory findings (Table 1) showed mild elevation of hepatobiliary enzymes but no elevation of tumor markers (i.e., carcinoembryonic antigen, carbohydrate antigen 19–9, and soluble interleukin-2 receptor [sIL-2R]). Contrast-enhanced computed tomography revealed mild intrahepatic bile duct dilatation, bile duct wall thickening in the perihilar region (Fig. 1a), and a 20-mm mass adjacent to the thickened bile duct (Fig. 1b). No other abnormal findings were observed in the imaging range from the chest to the pelvis. Magnetic resonance imaging revealed a thickened wall of the perihilar bile duct and the adjacent mass had a hyperintense signal on diffusion-weighted imaging (Fig. 2a). Magnetic resonance cholangiopancreatography revealed a stricture in the bile duct extending from the hepatic hilum to the vicinity of the confluence of the cystic duct, and the upstream bile duct was slightly dilated (Fig. 2b). Endoscopic ultrasonography revealed a thickened bile duct wall as much as 8-mm in diameter with smooth internal echoes (Fig. 3a) and a proper hepatic artery with a preserved lumen penetrating the wall (Fig. 3b). A 2-cm mass, found close to the lesion, was considered an enlarged lymph node. The boundary between the bile duct and mass was clear, and no continuity was observed (Fig. 3c). Endoscopic retrograde cholangiopancreatography (ERCP) revealed a relatively extensive and gently rising stricture in the perihilar bile duct (Fig. 4a). Meanwhile, intraductal ultrasonography showed homogeneous wall thickening (3.5 mm) in the stenotic area (Fig. 4b), but no wall thickening in the non-stenotic area (Fig. 4c). Peroral cholangioscopy (POCS) shows submucosal tumor-like-circumferential narrowing in the stenotic area, with mildly irregular and uneven but smooth mucosa, and no severe mucosal irregularities or unevenly dilated blood vessels suggestive of cholangiocarcinoma were observed (Fig. 4b). In the non-stenotic area, POCS reveals no mucosal irregularity (Fig. 4c). Therefore, transpapillary bile cytology, brush cytology, and forceps biopsy were performed; however, a pathologic diagnosis could not be made. Therefore, EUS-FNA was performed on the enlarged lymph nodes using the echoendoscope (GF-UCT260; Olympus Medical Systems, Tokyo, Japan) and a 22-G needle (EZ Shot 3 plus; Olympus Medical Systems). Specimens were obtained from the duodenal bulb by two punctures using a standard 10-ml suction method. Diffuse proliferation of large atypical cells with anisotropic and irregular nuclei was observed in the biopsy specimens (Fig. 5a). Immunohistochemical staining was positive for CD10, CD20, and BCL6, and negative for CD3 and MUM-1 (Fig. 5b–f). These findings confirmed germinal center B-cell-like diffuse large B-cell lymphoma (DLBCL), according to the Hans’ algorithm [8].

**Table 1:** Laboratory findings

Hematology		Biochemistry			
WBC	8700/μL	T-Bil	0.6 mg/dL	IgG	1017 mmol/L
RBC	496 × 10^4^/μL	AST	32 U/L	IgG4	43.1 mmol/L
Hb	14.9 g/dL	ALT	36 U/L	Na	136 mmol/L
Ht	44.7%	LDH	244 U/L	K	3.9 mmol/L
Plt	24 × 10^4^/μL	ALP 347	U/L	Cl	101 mmol/L
		r-GTP	106 U/L	CRP	0.16 mg/dL
Coagulation		AMY	76 U/L		
PT	124%	BUN	18.7 mg/dL	Tumor markers	
PT-INR	0.88	Cr	1.24 mg/dL	CEA	2.8 ng/mL
APTT	27.3 s	TP	7.1 g/dL	CA19-9	2 U/mL
		Alb	4.2 g/dL	sIL-2R	562 U/mL
		T-Chol	256 mg/dL		
		TG	76 mg/dL		

WBC, white blood cells; RBC, red blood cells; Hb, hemoglobin; Ht, hematocrit; Plt, platelets; PT, prothrombin time; PT-INR, prothrombin time-international normalized ratio; APTT, activated partial thromboplastin time; T-Bil, total bilirubin; AST, aspartate aminotransferase; ALT, alanine aminotransferase; LDH, lactate dehydrogenase; ALP, alkaline phosphatase; γ-GTP, γ-glutamyl transpeptidase; AMY, amylase; BUN, blood urea nitrogen; Cr, creatinine; TP, total protein; Alb, albumin; T-Chol, total cholesterol; TG, triglyceride; IgG, immunoglobulin G; IgG4, immunoglobulin G4; CRP, c-reactive protein; CEA, carcinoembryonic antigen; CA19-9, carbohydrate antigen 19–9; sIL-2R, soluble interleukin-2 receptor

Based on the diagnosis of DLBCL, the patient received six cycles of R-CHOP chemotherapy, comprising rituximab plus cyclophosphamide, doxorubicin, vincristine, and prednisolone, which resulted in complete remission. The abdominal pain resolved promptly after the initiation of treatment and was attributed to cholestasis caused by bile duct stricture. At 19 months post-diagnosis, the patient remained in complete remission (Fig. S1a, b).

## Discussion

Extranodal ML accounts for 20–40% of all cases [9]; the most common extranodal organs affected are along the gastrointestinal tract, including the stomach, small intestine, and colon [1]. Biliary stricture and obstructive jaundice have been reported in 1–2% of all cases, mostly as a result of compression of enlarged lymph nodes or invasion from adjacent organs [2]. Since its first report by Nguyen in 1982 [10], 52 cases of primary bile duct ML have been recorded [3, 5, 11–13], including our case. According to these reports, 11 cases were diagnosed without surgery (Table 2) [3, 11–19], while others were diagnosed preoperatively with cholangiocarcinoma and therefore underwent surgical resection. Among the 52 reported cases of bile duct ML including our case, results of sIL-2R levels were available in only seven cases. Four of these (57%) did not exhibit elevated sIL-2R levels, and all were limited to the bile duct [3, 12, 20]. The remaining three cases with elevated sIL-2R levels had disease in multiple organs [11, 17, 18]. Although the small number of cases makes it difficult to draw definite conclusions, elevated sIL-2R levels may indicate the spread of disease.

**Table 2:** Summary of reported cases of bile duct malignant lymphoma diagnosed without surgical resection

Case	Age/sex	Location	Tissue sampling methods	Pathologic diagnosis	Treatment	Outcome
Corbinais [14]	29/M	Common bile duct	ND	HGTL	COP plus CHOP	Alive for 12 months
Das [15]	51/M	Perihilar	US-guided needle biopsy	DLBCL	R-CHOP	Alive for 18 months
Torrealba [16]	69/M	Distal	Transpapillary biopsy (bile duct)	MALT lymphoma	Chemoradiation	Unknown
Ikuta [17]	66/M	Distal	Transpapillary biopsy (bile duct, major papilla)	MALT lymphoma	HPET R-CHOP	Recurrence at 12 months after HPET, PR after R-CHOP
Durham [13]	61/M	Perihilar	Transpapillary biopsy (bile duct)	HGBL	R-CHOP	Unknown
Inoue [18]	78/F	Perihilar	Transpapillary biopsy (bile duct)	MALT lymphoma	R-CVP	CR for 12 months
Ohtsubo [11]	67/F	Perihilar	Transpapillary biopsy (bile duct, major papilla)	HGBL	R-CHOP DA-EPOCH-R	CR for 18 months
Pararas [19]	61/F	Perihilar	Diagnostic laparoscopy	DLBCL	R-CHOP	No recurrence for 8 months
Sakashita [3]	80/F	Perihilar	Transpapillary biopsy (bile duct)	MALT lymphoma	Rituximab	No recurrence for 12 months
Imai [12]	54/M	Distal	Transpapillary biopsy, EUS-FNA (bile duct)	DLBCL	R-CHOP	No recurrence for 10 months
Present case	70/F	Perihilar	EUS-FNA (lymph node)	DLBCL	R-CHOP	CR for 19 months

M, male; F, female; US, ultrasound; EUS-FNA, endoscopic ultrasound-fine needle aspiration; HGTL, high grade T-cell lymphoma; DLBCL, diffuse large B-cell lymphoma; MALT, mucosa-associated lymphoid tissue; HGBL, high grade B-cell lymphoma; COP, cyclophosphamide, oncovin and prednisone; R-CHOP, rituximab, cyclophosphamide, doxorubicin, vincristine, and prednisone; HPET, *Helicobacter pylori*-eradication therapy; R-CVP, rituximab, cyclophosphamide, vincristine, and prednisone; DA-EPOCH-R rituximab, etoposide, prednisolone, vincristine, cyclophosphamide and doxorubicin; CR, complete remission, PR partial remission

Accurate diagnosis of bile duct ML is difficult, in part because imaging characteristics of bile duct ML are not well-known. Yoon et al. [4] reported that the characteristic findings of primary bile duct ML were smooth, mild luminal narrowing of the extrahepatic bile duct without mucosal irregularity, despite diffuse thickening of the bile duct wall. In addition cholangioscopic findings of bile duct ML have only been reported in five cases, including ours [3, 12, 16, 20]. Three lesions in the two cases of MALT lymphoma exhibited a nodular lesion with ulceration and increased vascularity, flat lesion, and flat-depressed lesion with increased vascularity. In contrast, three patients with DLBCL presented with submucosal tumor-like lesions showing circumferential narrowing, with no abnormal findings on the mucosal surface. These characteristics are continuous with those of primary gastrointestinal ML [21]. Moreover, intestinal DLBCL, the most common histologic type of NHL, presents with a long circumferential stricture, while luminal extension is preserved, and passage is not easily impaired [22]. The present case of DLBCL also presented a smooth stricture, which was mild compared with the degree of wall thickening. As these findings resemble those of IgG4-related sclerosing cholangitis (IgG4-SC) rather than cholangiocarcinoma, bile duct ML may also be considered in the differential diagnosis when IgG4-SC is suspected. The characteristic POCS findings of the stricture site of IgG4-SC are a smooth mucosal surface, lack of easy bleeding, and dilated tortuous vessels with less abrupt caliber alteration and disruption than those in extrahepatic cholangiocarcinoma [23]. Our case concurs with the mucosal and bleeding findings but differs in the presence of dilated blood vessels, which may indicate a point of differentiation between bile duct ML and IgG4-SC; however, as there are few reported cases, further studies are warranted. Cholangiocarcinoma and primary sclerosing cholangitis (PSC) are also important in the differentiation of bile duct stricture. POCS findings of cholangiocarcinoma is characterized by irregularly dilated and tortuous vessels and an irregular papillrogranular surface [24], while POCS findings of PSC is characterized by scarring and pseudodiverticula [25]. In the present case, there was mild irregularity of the mucosa of the stricture, but no dilated vessels were present, nor were there any findings suggestive of PSC.

Another reason for the difficulty in diagnosing bile duct ML is the limited collection of pathologic samples. Brush cytology with ERCP and transpapillary forceps biopsies are routinely used to diagnose biliary strictures. However, the sensitivities of brush cytology, forceps biopsy, brush cytology, and forceps biopsy were poor, at 41%, 52%, and 66%, respectively [26], and many cases required surgery for the diagnosis of cholangiocarcinoma. In this case, a diagnosis could not be made by transpapillary biopsy. Although some cases of ML have been diagnosed through transpapillary biopsy (Table 2), many cases of bile duct ML have been misdiagnosed as cholangiocarcinoma, leading to unnecessary surgery. This may be due to the submucosal location of lesions, as observed with POCS, making it difficult to obtain adequate samples through forceps biopsy. Recent reports highlight the usefulness of EUS-FNA in improving the diagnostic performance of bile duct lesions [26–28]. The sensitivity and specificity of EUS-FNA for bile duct lesions not diagnosed by ERCP have been reported to be 77–89% and 100%, respectively [27], and for enlarged lymph nodes, 94% and 100%, respectively [28]. However, EUS-FNA of bile duct lesions poses potential risks, including biliary fistulas formation and needle-tract seeding [29], warranting careful consideration before its application. In cases of bile duct strictures where transpapillary biopsy fails to provide a diagnosis, as in the present case, EUS-FNA targeting enlarged, accessible lymph nodes may be prioritized. Conversely, one case of bile duct ML has been reported wherein repeated transpapillary biopsy via ERCP and EUS-FNA, successfully confirmed bile duct ML [12]. Thus, when a false-negative result is suspected, a short-term re-examination with transpapillary biopsy or EUS-FNA should be considered.

In the present study, we encountered a case of ML of the extrahepatic bile duct, which had been diagnosed via detailed imaging examination and EUS-FNA. Diagnosing bile duct ML is challenging, and failure to achieve a diagnosis may necessitate highly invasive procedures, such as pancreaticoduodenectomy or hepatectomy. Utilizing both transpapillary biopsy and EUS-FNA, when necessary for a definitive diagnosis, could help prevent unnecessary surgeries and offer considerable benefits for patient outcomes. Our findings suggest that, when relatively extensive submucosal wall thickening and mild luminal narrowing without mucosal surface irregularities mimicking IgG4-SC are present, ML should be considered in the differential diagnosis. In addition, our report supports that POCS and EUS-FNA can contribute to an accurate diagnosis of bile duct ML.

## Supplementary Information

Below is the link to the electronic supplementary material.

**Fig.** **S1** Image findings 19 months after initiation of treatment. a The bile duct wall thickening is improved in plane CT. b No significant 18-fluorodeoxyglucose uptake is observed. (JPG 1947 KB)
