# Supplementary figures and images for: A case of malignant lymphoma of the extrahepatic bile duct diagnosed by detailed imaging examination and endoscopic ultrasound-guided fine needle aspiration
Source: Clin J Gastroenterol. 2024 Nov 30;18(1):176–82. doi: 10.1007/s12328-024-02075-x (PMC11785620; doi:10.1007/s12328-024-02075-x)

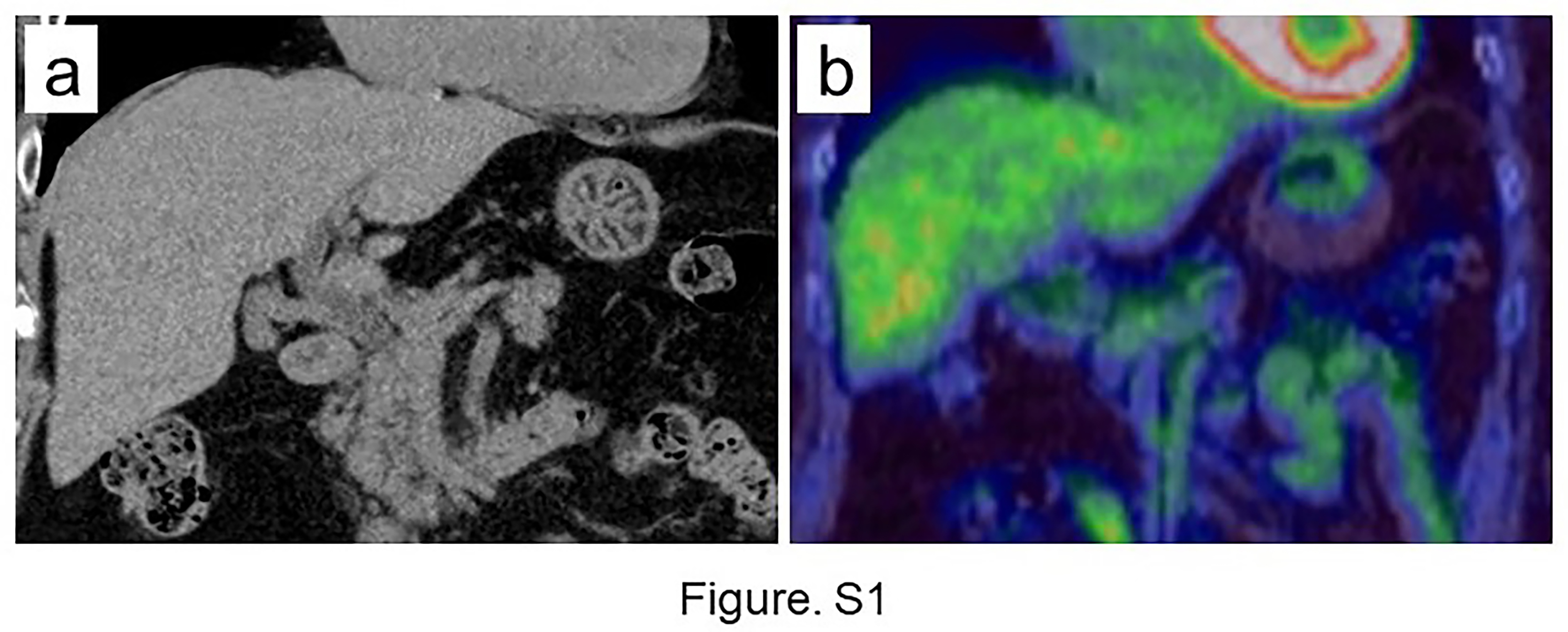

Supplement: Supplementary file 1 — Fig. S1 Image findings 19 months after initiation of treatment. a The bile duct wall thickening is improved in plane CT. b No significant 18-fluorodeoxyglucose uptake is observed. (JPG 1947 KB) [file 12328_2024_2075_MOESM1_ESM.jpg]
